# Genetic counselors and Genomic Counseling in the United Kingdom

**DOI:** 10.1002/mgg3.123

**Published:** 2014-12-09

**Authors:** Anna Middleton, Georgina Hall, Christine Patch

**Affiliations:** 1 Wellcome Trust Sanger InstituteCambridge, United Kingdom; 2 Manchester Centre for Genomic Medicine, Central Manchester University Hospitals NHS Foundation Trust, Manchester Academic Health Sciences Centre (MAHSC), St. Mary's HospitalManchester, United Kingdom; 3 Guys and St Thomas' NHS Foundation TrustLondon, United Kingdom; 4 Kings College LondonLondon, United Kingdom

## Introduction

Mainstream medical services, whether they are oncology, pediatrics, infectious diseases, cardiology, or dermatology are increasingly using genomic data in patient management (Graham 2007; Feramisco et al. 2010; Padmanabhan et al. 2013; Mantzouranis et al. 2014; Rahman 2014). Through the 100,000 Genomes Project in the United Kingdom, 100,000 genomes will be sequenced by 2017 in the National Health Service (www.genomicsengland.co.uk). Health Education England (HEE) is responsible to the UK government for creating a lasting education legacy to support thousands of healthcare professionals as they upskill in response to this genomic revolution. The place of genetic counselors, genetic counseling, and ultimately “genomic counseling” within this mix forms the focus of this Commentary. This piece is written with the British context in mind, where genetic counseling is delivered predominantly within regional clinical genetics services by the government-funded National Health Service (NHS).

## Genetic Counseling: The Emotional Context is Integral

“Genetic counseling is conducted by healthcare professionals who have been specially trained in the science of human genetics (a genetic counselor or a clinical geneticist)” (NHS Choices 2014). The word “counseling” refers to information-giving or communication as opposed to psychotherapy or psychology thus *doing* genetic counseling means communicating genetic information in a meaningful way to patients. But it does not just mean having a conversation that happens to involve genetic information; it goes much further than that. Genetic counseling is a *process* of “helping people understand and adapt to the medical, psychological and familial implications of genetic contributions to disease” (Resta et al. 2006); it involves a person-centered approach (Hough 2002) where the genetic counselor helps the patient to incorporate the genetic information into their lives, adjust to it, rationalize it, think through how they want to act on it and rehearse how they wish to explain it to relatives.

Genetic counseling is frequently an emotional process (McCarthy Veach et al. 2003a); the patient may come for genetic counseling at one of the most vulnerable moments in their life, an event caused by a faulty gene may result in grief (“my husband has suddenly died”), loss (“my child has a neurodegenerative condition”), or crippling fear (“I'm frightened I will develop the cancer running through my family.”) Helping patients in that moment, and being emotionally congruent (McCarthy Veach et al. 2003b), as they discuss the impact of the genetic event, is at the heart of what genetic counselors and clinical geneticists do.

In many cases, a referral for genetic counseling is prompted by the presence of a family history of a condition associated with a highly penetrant Mendelian condition, for example, Duchenne Muscular Dystrophy, cystic fibrosis, Huntington's Disease. Interlinked with this is often a strong emotional context, where patients have experienced the condition in their family and have well informed ideas about what it means to live with the condition or be at risk of developing it. A conversation then happens with the patient about what this genetic information means to them and how they want to use it. The emotional place the condition has in the family can be very much part of this conversation. A discussion will also occur about other relatives at risk of the condition in the family and how to pass on information to them.

In the context of a family history, genetic counselors, and clinical geneticists traditionally have worked from the phenotype back to the genotype and defined, through genetic analysis, whether there is a single gene fault that is responsible for the family condition. The emotional connection to a particular condition, which is provided by the lived-experience of it, helps to guide the patient in their decisions, for example, to give informed consent for genetic testing. This is strikingly different from a patient who (seemingly) has no family history of a particular condition who is then faced with a genome-based test and subsequent result (Middleton 2012). Taking informed consent for such testing and supporting patients with the results (as emotionally challenging they may or may not be) is likely to be at the forefront of genomic counseling.

“Genetic counselor” is an internationally recognized job title that defines a specific group of health professionals with recognized qualifications, training, and registered/certified competency to practice (Skirton et al. 2013). Worldwide, genetic counselors are often trained through Masters programmes in the theory and delivery of genetic counseling and have established core competencies to practice (e.g., see www.gcrb.org) and outcome measures to define success (McAllister et al. 2011).

## A Distinction Between Genetic Counseling Done by Clinical Geneticists and Genetic Counselors

Primarily, clinical geneticists are tasked with the diagnosis and clinical management of patients with genetic disease. Whilst both clinical geneticists and genetic counselors use skills in communication and information provision, genetic counselors have specific training in how to deliver psychosocial support to patients. Thus, once a diagnosis is clear, the genetic counselor focuses her or his work on explaining patterns of inheritance, exploring how this impacts on the patient and their family, helping the patient to manage the burden of disease, navigate the challenges of autonomous decision making within the context of family dynamics and support family communication.

Within the realm of genomic medicine, after a sequence is performed, interpreting the significance of a variant and making an accurate clinical diagnosis requires the skills of a multidisciplinary team of scientists, bioinformaticians, and clinicians. In the UK currently, the clinical geneticist is more likely to be involved in this than a genetic counselor. However, in some specialist clinics already the genetic counselor is participating in variant interpretation. This is true in the US too (Ormond 2013).

## A Distinction Between Genetic Counseling Done by Other Health Professionals and Genetic Counselors

Within the clinical genetics setting, the act of *doing* genetic counseling is used quite liberally to describe the process that both clinical geneticists and genetic counselors attend to, but other health professionals have also adopted this verb. For example, an obstetrician who does chorion villus sampling (CVS) may need to explain a genetic test result (before or instead of a referral to clinical genetics). In this situation they are unlikely to explore the implications of the result for family members nor be arranging cascade screening, however, they are likely to be using strong, empathic communication skills to help the patient understand and appreciate the significance of the genetic information (Arnold and Self 2012). We would say that aspects of genetic counseling practice are being used here. Whether this should be termed “having a conversation based on genetic information” or “genetic counseling” (as per the descriptions as highlighted above) is open for debate.

A key distinction between the current conversations had about genetics/genomics in mainstream medicine and the work within clinical genetics services is that primarily nonclinical genetics healthcare is based on an individual and clinical genetics healthcare is based on a family. For example, a pediatrician seeing a child with developmental disorder will focus on the delivery of diagnosis and care for that individual child. Whereas a clinical geneticist seeing the same child would extend their care to the family, exploring risks of developmental disorder in siblings, and other relatives. Thus, when mainstreaming genomics across a whole health service, it is pivotal that decisions are made as to what, if any, family care will be covered outside of clinical genetics.

## Genomic Medicine and Genomic Counseling

As the discipline of genomics is so new, there has not been time yet to establish consistent and internationally recognized definitions of the terms “genomics,” “genomic medicine,” and “genomic counseling;” and these terms are often used interchangeably and in different ways by health professionals and scientists. What is not clear is whether “genomic counseling” is simply any healthcare scenario that deals with data derived from a genomic sequencing assay or whether it is an extension of genetic counseling (or maybe even both). “Debate about what ‘genomic counseling’ will include and who will practice it has been fueled by the transition from single-gene focused genetic counseling and testing to a full genomic medicine approach” (Ormond 2013, p. 189).

In terms of patient management, the breadth and scope of genomic data to be revealed from a sequenced assay, is potentially enormous. Metaphorically speaking, when searching a genomic sequence (library) for a particular gene(s) (book) we may: (1) search for one individual book or select books on one topic (e.g., use the genome as a resource from which to select one highly penetrant cancer gene or a panel of cancer genes, for further in-depth analysis); or (2) choose to browse all the shelves and pick out a collection of books across multiple topics (e.g., a screen of a healthy person, embryo or fetus to predict disease in the future).

Each of these scenarios is described in more detail:

## Genomics as a Resource for Highly Penetrant, Single-Gene Data

If a whole-genome sequence is done to answer a specific clinical question, for example, why was my child born blind? The sequence is used as a resource of DNA, but a specific clinical question can be answered via a targeted bioinformatics analysis, for example, only looking at a panel of visual impairment genes. Here, the clinical question is specific and despite genomic technologies being used as part of the testing repertoire, as the results are targeted; it is almost insignificant that the mechanism to obtain the result uses sequencing technology. From the genetic counselor's perspective; the experience of delivering the results to the patient is the same. Here, there may be a strong emotional context, with the necessity to support multiple members of the family to communicate and evaluate risk of developing the same condition. Again genomic counseling (if we were to call it this here) is really just the same as genetic counseling.

**Figure 1:**
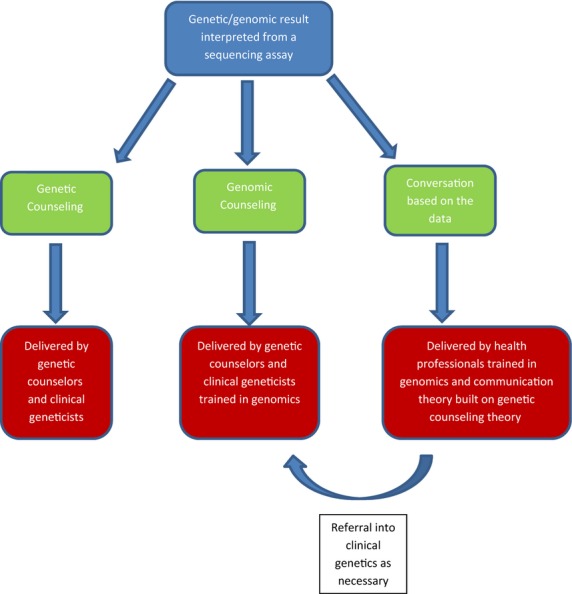
Potential models for communicating genomic data.

What is pivotal in terms of education is that health professionals in mainstream medicine understand that not all genes are deterministic. In this interim period while genomic technologies are being introduced into mainstream services, but the training in interpretation has not yet caught up, the potential for inappropriate referrals into clinical genetics services is enormous. For example, anecdote from colleagues in clinical genetics suggest a significant increase in a new wave of referrals for common polymorphisms, such genomic results may not be significant in terms of health and do not require the involvement of a clinical geneticist or genetic counselor. As education about genomics filters into mainstream practice, such a result can be handled appropriately without specialist referral (Fig.1).

## Genomics as a Screening Tool that Predicts Future Disease

Depending on what question is being answered through the screening, a whole-genome/exome sequence would provide multiple options for output, ranging from a few results to potentially hundreds or even thousands. In making these choices, we need to be cognizant of the limitations in our ability to interpret much of the rare variation present in our genomes and the ascertainment bias (knowledge is derived largely from the study of affected individuals) that confounds the use of much of our existing knowledge in a screening context.

Given the limitations in interpretation, should a screening mechanism be chosen, it could be applied in several different ways. For example, screening could be done opportunistically each time a sequence is performed for specific clinical reasons, for example, when offering an exome sequence for a child with developmental disorders an opportunistic screen for 24 cancer and cardiac conditions could be performed at the same time (Green et al. 2013). Or it could be applied as the first-line test, for example, in a prenatal setting on cell-free DNA to check the baby for abnormalities (Fan et al. 2012).

In such a setting clinicians aim to predict future health (phenotype) based on a genotype, when the phenotype does not exist yet (i.e., this is the opposite approach to what has historically happened within a clinical genetics service). Communicating risk and possibly multiple risks containing multifactorial effects and helping the patient to adapt and incorporate this could constitute ‘genomic counseling’ in this scenario.

If the screening is being used in a healthy adult as part of a public health agenda, let's assume the patient has no awareness of, or concerns about, any particular conditions in their family. Due to this there may be limited emotional triggers, pretest, to guide and inform the patient about the conditions being tested for (Middleton 2012). Autonomous decision making here, with respect to consent, is different to situations frequently encountered within genetic counseling where there are strong emotional cues to guide patients. If a patient has no personal connection or any knowledge of any of the conditions being tested for, then extensive pretest counseling is unlikely to be meaningful. It is also not practical to explain the significance of multiple variants, pretest, and have a lengthy discussion about each. Therefore, existing models of consent, as used in current genetic counseling clinics, will need to evolve. In turn existing models of results delivery will also need to evolve as we learn the implications of an unfavorable result, or series of results, that arrive with limited emotional preparation for patients, who have not had extensive pretest counseling.

## Genomic Counseling and Genetic Counselors

If one was to assume that genomic counseling is simply the management of data revealed through whole-genome analytic approaches – which could result in single-gene data through to multiple gene data – then it seems a natural progression for genetic counselors to upskill their genetic knowledge into genomic knowledge and evolve their practice. It is not known yet how and whether the same communication theory will apply to the genomic era; but existing genetic counseling models may be used as a strong starting point. New theories of communication of genomic information may develop that support the delivery of multiple results (Hooker et al. 2014).

Genomic information may be delivered without the emotional conversation – it is not necessary, (as well as being impossible on a practical level), for genetic counselors to gate-keep genomic information delivery for mainstream medicine. They may, however, have a large role in supporting other healthcare practitioners to do so. There will be many conversations involving genomic data that have limited emotional context, for these, all that is required is a health professional who understands how to interpret the data and incorporate this into their usual practice. However, if ever the receipt of genomic data does result in a more complex reaction (e.g., “how is this relevant to my external family” or “I don't know how to cope with this”) then referral into a clinical genetics service is still recommended.

Clinical geneticists and genetic counselors manage whole families rather than individuals, work with the emotional fallout from inherited disease and are experts in rare multisystem and inherited genetic conditions and so it could be argued that there will always be a need for this specialist domain of care.

## The Genomic Counselor

It is not clear whether genetic counselors internationally should lay claim to the title “genomic counselor.” However, what is clear is that genomic medicine is being mainstreamed on an enormous scale and that health professionals who have no connection to clinical genetics and who have no understanding of what genetic counselors do, will be delivering information gleaned from genomic technology. The HEE have recognized that there is an urgent need to address training within the NHS to upskill the workforce with respect to genomic knowledge. What is necessary to develop alongside this is an upskilling of knowledge in how this information can be communicated and the implications for families. Genetic counselors are in a strong position to inform this teaching.

If we decide that “genomic counseling” is the act of providing information about genomic data (with or without some level of emotional context) then it is logical to conclude that through the upskilling of the workforce, most health professionals in the NHS will be *doing* some level of genomic counseling; but, does that make them a “genomic counselor?” Decisions will have to be made about whether this remains the domain of genetic counselors. Our sense is that genetic counselors and clinical geneticists will become expert practitioners in genomic counseling and will specialize in the management of genomic information delivery when there is an emotional and familial context (“Specialist Genomic Counselors”). “Genetic counselors have a long-standing history of working on the clinical forefront of implementing new genetic technology. Genomic sequencing is no exception” (Hooker et al. 2014, p. 445). Other health professionals will have “conversations based on genomic data” as part of their normal healthcare delivery, but they would not define themselves as a genomic counselor without some level of training in communication theory bourn from genetic counseling. A genomic counselor, like genetic counselor, would also presumably work with the wider family to explore the implications of any genomic test result for relatives.

In time a new hierarchy may be established, linked to qualifications and training and a distinct profession may emerge with protection over the title “genomic counselor.” In the meantime, genetic counselors need to engage with the debate about this, upskill their own knowledge with respect to bioinformatics, sequencing, data interpretation, and visualization and the ethical, legal, and social issues raised by genomics and in time, decide whether they will lead or be led with respect to genomic counseling.
